# Case of HER2neu+ Invasive Pleomorphic Lobular Carcinoma With Response to Conventional Neoadjuvant Chemotherapy: A Viable Option for an Exceedingly Rare Breast Cancer Type

**DOI:** 10.7759/cureus.11108

**Published:** 2020-10-23

**Authors:** Blessie Nelson, Ashley M Brizendine, Rachelle Gietzen, Rohit Venkatesan

**Affiliations:** 1 Department of Hematology and Medical Oncology, University of Texas Medical Branch, Galveston, USA; 2 Department of Pathology, University of Texas Medical Branch, Galveston, USA; 3 Department of Hematology and Oncology, University of Texas MD Anderson Cancer Center, Galveston, USA

**Keywords:** tchp, neoadjuvant chemotherapy, invasive pleomorphic lobular breast carcinoma

## Abstract

Invasive pleomorphic lobular carcinoma (IPLC) is an extremely rare form of breast cancer that accounts for less than 1% of all breast cancer cases. Due to this rarity, currently, there is a lack of an established standard of care for patients diagnosed with this form of breast cancer. In this case report, we present a 57-year-old female with a complex oncologic history diagnosed with clinical prognostic Stage IIA (ER 5%, PR 0%, HER2neu 3+) invasive pleomorphic lobular carcinoma of the left breast treated with neoadjuvant docetaxel, carboplatin, trastuzumab, and pertuzumab-based therapy (TCHP) followed by surgery. Surgical pathology revealed treatment-related changes with a definite response to neoadjuvant therapy. We report this case to highlight the response of this rare pathological entity to a standard neoadjuvant regimen such as docetaxel, carboplatin, trastuzumab, and pertuzumab.

## Introduction

Breast cancer is a devastating diagnosis that has disrupted the lives of millions of women across America. New incidences of female breast cancer occur at a rate of 127.5 per 100,000 women per year and constitute 15.2% of all new cancer cases [1]. Despite its frequent diagnosis, breast cancer is a heterogeneous group comprising a variety of subtypes of which the most common subtype is invasive ductal carcinoma comprising 80% of breast cancer cases [2]. Other subtypes are rarer, such as invasive lobular carcinoma, which accounts for 5%-15% of breast cancer cases [3]. Of these cases, only 15% are invasive pleomorphic lobular carcinoma (IPLC) resulting in invasive pleomorphic lobular breast cancer accounting for approximately 1% of total breast cancer cases [4]. When further analyzed, 0%-81% of invasive pleomorphic lobular breast cancer overexpress the proto-oncogene HER2 hence demonstrating its variability among different studies [5]. Due to this rarity, HER2-positive invasive pleomorphic lobular breast cancer are sparsely documented in the literature and lacks a defined standard of care. Treatment options are generally determined by extrapolating from data generated from invasive ductal carcinoma. The goal of this case report is to assess the pathological response of HER2-positive invasive pleomorphic lobular breast cancer to neoadjuvant docetaxel, carboplatin, trastuzumab, and pertuzumab therapy, which demonstrated a 52% pathological complete response rate and no increased risk of cardiac toxicity in the TRYPHAENA trial in non-pleomorphic subtypes [6]. The response of invasive pleomorphic lobular breast cancer to neoadjuvant chemotherapy with a standard dual HER2neu blockade has been reported only sparingly in the literature.

## Case presentation

**Figure 1:**
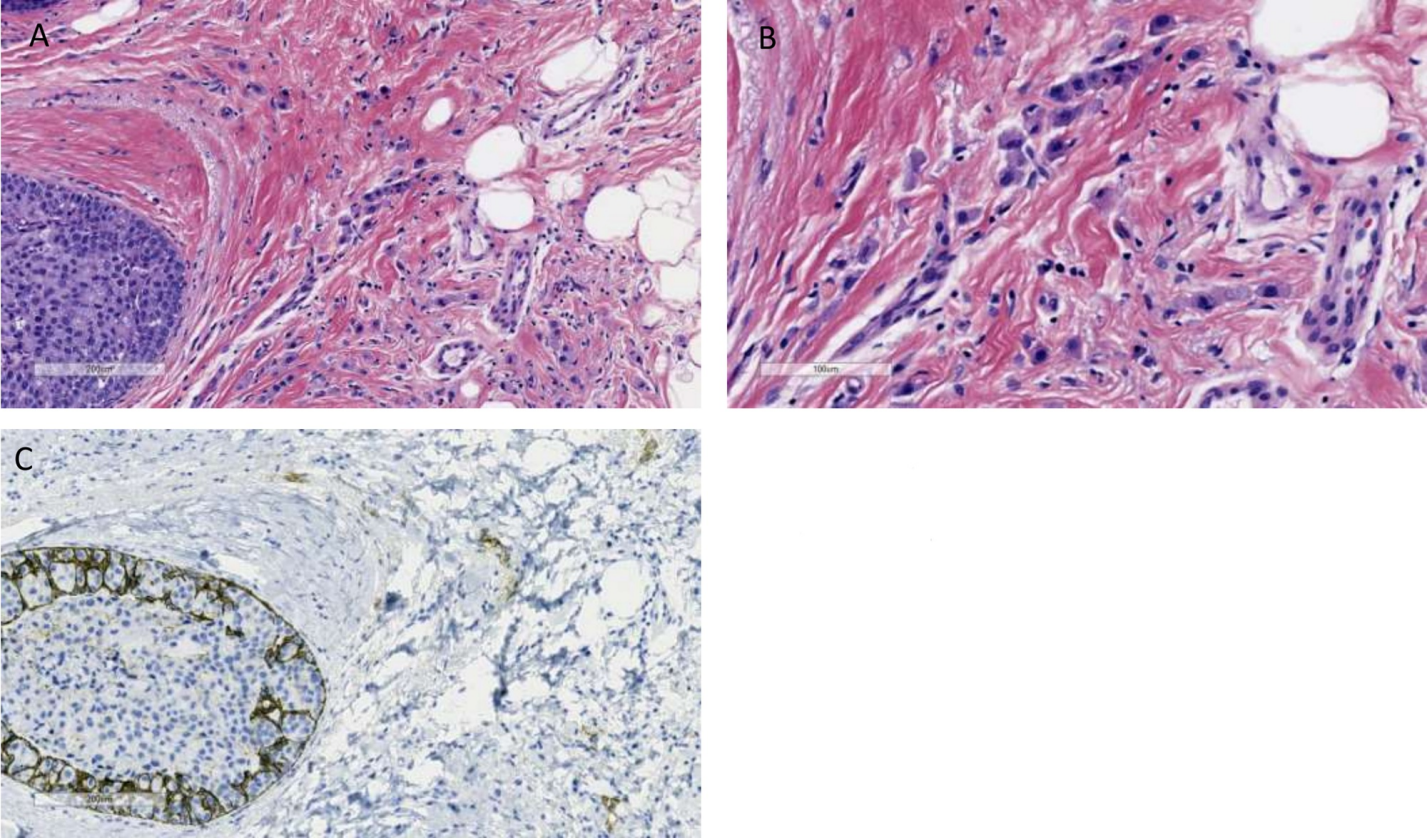
Initial biopsy: microscopic morphology of invasive and in-situ pleomorphic lobular carcinoma (A) at low power (x100) and (B) at high power (x200) with H&E staining. The malignant cells are arranged in a single file and show enlarged nuclei and abundant eosinophilic granular cytoplasm. (C) E-cadherin staining, negative in neoplastic cells (×100). H&E: hematoxylin and eosin

**Figure 2:**
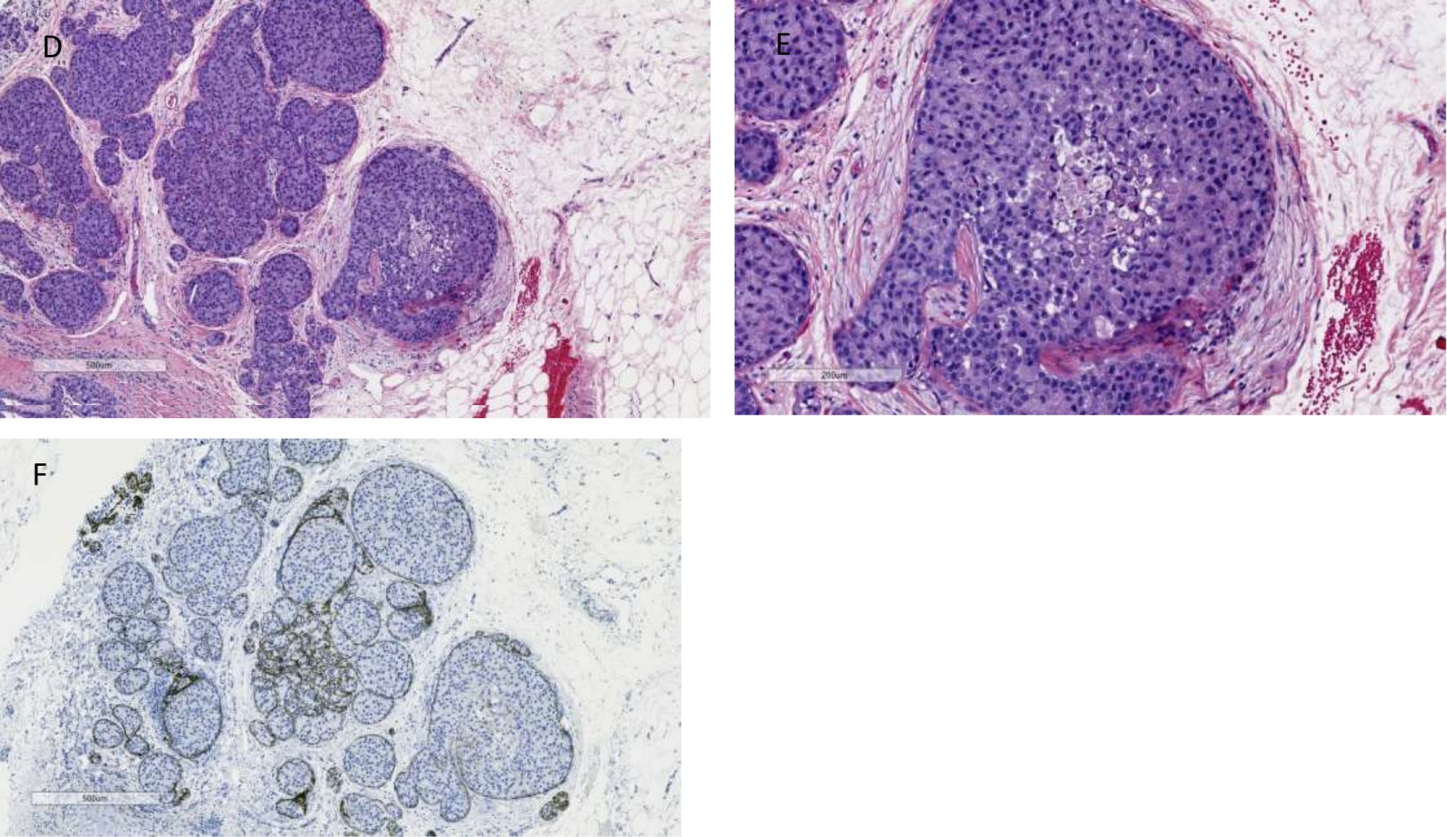
Initial biopsy: this section shows lobular carcinoma in situ (LCIS), pleomorphic variant with signet ring cell changes (D) at low power (x40) and (E) at high power (x100) with H&E staining. (F) LCIS image with E-cadherin staining, neoplastic cells negative for E-cadherin (×40). H&E: hematoxylin and eosin

**Figure 3:**
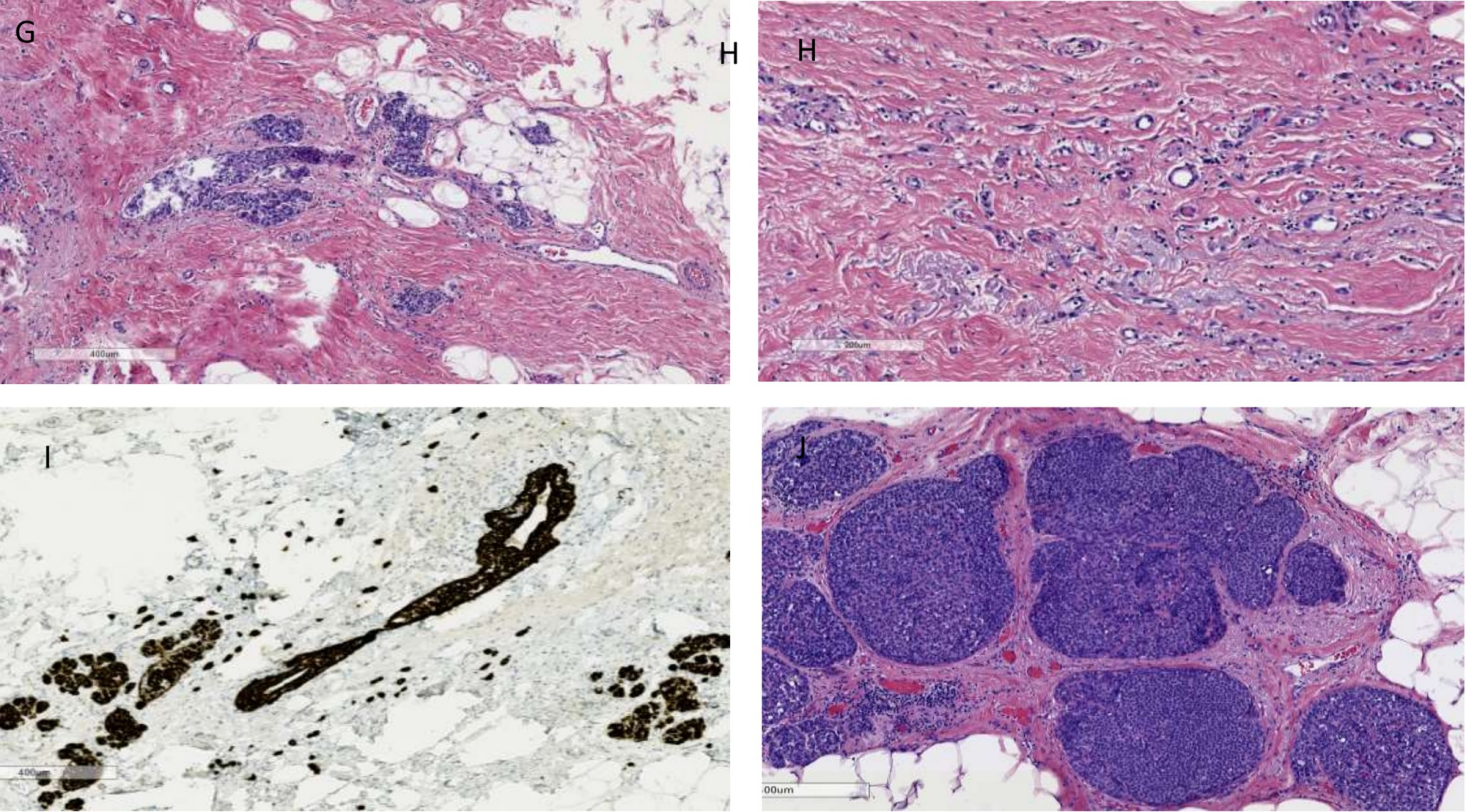
Post chemotherapy breast resection: invasive pleomorphic lobular carcinoma (IPLC) with few residual cells identified within the tumor bed (G) at low power (x50) and (H) at high power (x100) with H&E staining showing treatment effect. (I) LCIS image with cytokeratin 7 staining showing few residual neoplastic cells seen (×50). (J) Image showing pleomorphic and classic residual LCIS with treatment response (×50) H&E: hematoxylin and eosin; LCIS: lobular carcinoma in situ

This is a case of a 57-year-old Caucasian female who, in September 2019, presented for her annual screening mammogram, which revealed grouped 35 mm x 23 mm x 17 mm coarse heterogenous calcifications in the left breast. Ultrasound-guided biopsy of the mass revealed pleomorphic invasive lobular carcinoma with a signet cell component, which was estrogen receptor (ER) (5%), progesterone receptor (PR) (0%), and HER2neu 3+ positivity by immunohistochemistry (IHC) as seen in Figures 1A-1C and Figures 2D-2F. The patient also underwent genetic screening, which was negative. She went on to receive neoadjuvant chemotherapy with docetaxel, carboplatin, trastuzumab, and pertuzumab for six cycles followed by surgical resection. She tolerated chemotherapy well with expected toxicities and did not require hospitalization during treatment. After completion of neoadjuvant chemotherapy, she underwent a segmental mastectomy of the left breast with sentinel lymph node mapping of the left axilla and oncoplastic reconstruction with axillary nodal dissection. The entire tumor was measured to be 2.4 x 1.2 cm and was composed of invasive and in-situ lobular carcinoma foci that involved 4% of the tumor bed, as seen in Figures 3G-3J. Treatment-related changes were seen with a definite response as per the College of American Pathologists (CAP) guidelines. Margins were negative for invasive carcinoma and no lymphovascular invasion was identified. Of the three sentinel lymph nodes removed, macrometastatic (5 mm) lobular carcinoma was identified in one sentinel node and five isolated tumor cells were identified in the third sentinel node. The remaining 15 lymph nodes dissected from the axilla were all negative for metastatic carcinoma. Pathological staging was noted as ypT1a(m) ypN1a pMNA. Due to the residual invasive pleomorphic lobular carcinoma, the patient is currently undergoing trastuzumab emtansine (Kadcyla) for 14 cycles adjuvantly per the KATHERINE trial [7].

## Discussion

Invasive lobular carcinoma is characterized by the loss of E-cadherin resulting in discohesive growth with the characteristic histologic appearance of cells growing in the single file pattern. Invasive pleomorphic lobular breast cancer is a histologically distinct subtype of invasive lobular cancer first described in 1982 by Dixon et al. and officially recognized by the World Health Organization (WHO) in 2003. In addition to the loss of E-cadherin, invasive pleomorphic lobular breast cancer demonstrates “increased nuclear size, nuclear pleomorphism, nucleolar prominence, and increased mitotic activity” [8-9].

In a retrospective study conducted by Hatch et al., 163 cases of non-pleomorphic invasive lobular carcinoma (NPL) were compared to 35 cases of invasive pleomorphic lobular breast cancers. The study demonstrated that invasive pleomorphic lobular breast cancer more often presented with a higher stage and metastatic disease at initial diagnosis. Of the invasive pleomorphic lobular breast cancer cases studied, 1/3rd presented at stage III-IV and 17.6% presented with metastases compared to only 1/5th and 7.7% of NPL cases, respectively. Interestingly, progression-free survival (PFS) was higher in invasive pleomorphic lobular breast cancer cases at 30% compared to 21% of NPL cases but the five-year overall survival (OS) rate was lower at 68.5% for invasive pleomorphic lobular breast cancer compared to 83.9% of NPL [10].

The difficulty of determining a standard of care for invasive pleomorphic lobular breast cancer is the rarity of the cancer itself. Because invasive pleomorphic lobular breast cancers constitute less than 1% of total breast cancer cases, large-scale clinical trials assessing the response of invasive pleomorphic lobular breast cancers to various treatment regimens is implausible. Well-powered clinical trials, such as the TRYPHENA and KATHRINE trials, consisted of patients with invasive ductal carcinoma (IDC). This resulted in treatment options for rare forms of breast cancer extrapolated from the results of the above-published trials. It is pertinent to note that invasive pleomorphic and lobular breast cancer have clinicopathologic characteristics distinct from invasive ductal cancer. For instance, it has been hypothesized that due to the loss of E-cadherin, decreased cell-to-cell adhesion facilitates greater local and metastatic spread of invasive lobular cancer as compared to invasive ductal cancer [11]. In one study, the rate of multiple metastases in invasive lobular cancer was 25% as compared to only 15.8% in invasive ductal cancer with more metastatic lesions detected in the bone, peritoneum, and a variety of other sites [12]. In a retrospective study conducted by Jung et. al., 35 patients with invasive pleomorphic lobular breast cancers were compared to 6,184 patients with invasive ductal cancer not otherwise specified to determine the clinicopathologic characteristics and prognostic differences between the two groups. Clinicopathologic characteristics identified in this study included invasive pleomorphic lobular breast cancers presenting with larger tumor size and poorer histologic grade, as well as higher rates of being staged at N3, nipple-areolar complex involvement, axillary node metastasis, and multifocal/multicentric involvement, resulting in mastectomy when compared to invasive ductal cancer [13]. During the follow-up period of 36 months, 14.3% of patients with invasive pleomorphic lobular breast cancers experienced disease recurrence, and 8.6% experienced disease-specific mortality as compared with 10.4% and 5.4%, respectively, in the invasive ductal cancer group. Despite being unable to directly link invasive pleomorphic lobular breast cancers' histology with a poorer prognosis, they were able to demonstrate the clinicopathologic characteristics, which account for its more aggressive nature when compared to invasive ductal cancer [13].

For our patient, after completing six cycles of docetaxel, carboplatin, trastuzumab, and pertuzumab therapy, surgical pathology revealed that there were very few scattered foci of residual tumor cells accounting for <2% of the tumor bed. Despite the well-documented natural history, aggressive biology, clinical course, and poor outcomes of the rare invasive pleomorphic lobular breast cancer entity, our patient had a definite treatment response pathologically to standard neoadjuvant therapy.

## Conclusions

Due to the differences in clinicopathologic characteristics and the more aggressive nature of invasive pleomorphic lobular carcinoma, larger randomized studies focused on invasive pleomorphic lobular breast cancers are necessary to determine the optimal standard of care for patients diagnosed with this rare form of breast cancer. Evidence in targeting this entity with documented responses to neoadjuvant chemotherapy with docetaxel, carboplatin, and dual HER2neu blockade is limited to sparse retrospective studies and case series. We present this case to add an additional illustration of a good response to neoadjuvant chemotherapy, which is an appropriate and viable approach when definite guidelines are deficient in managing invasive pleomorphic lobular breast cancers.
